# Understanding the impact of lumbar disc degeneration and chronic low back pain: A cross-sectional electromyographic analysis of postural strategy during predicted and unpredicted postural perturbations

**DOI:** 10.1371/journal.pone.0249308

**Published:** 2021-04-01

**Authors:** Janet A. Deane, Adrian K. P. Lim, Alison H. McGregor, Paul H. Strutton

**Affiliations:** 1 Department of Surgery and Cancer, Imperial College London, London, United Kingdom; 2 Department of Health Sciences, The University of Manchester and Manchester University NHS Foundation Trust, Manchester, United Kingdom; 3 Department of Imaging, Imperial College Healthcare NHS Trust, London, United Kingdom; Universita degli Studi di Roma ’Foro Italico’, ITALY

## Abstract

People with chronic low back pain (LBP) exhibit changes in postural control. Stereotypical muscle activations resulting from external perturbations include anticipatory (APAs) and compensatory (CPAs) postural adjustments. The aim and objective of this study was to determine differences in postural control strategies (peak amplitude, APAs and CPAs) between symptomatic and asymptomatic adults with and without Lumbar Disc Degeneration (LDD) using surface electromyography during forward postural perturbation. Ninety-seven subjects participated in the study (mean age 50 years (SD 12)). 3T MRI was used to acquire T2 weighted images (L1-S1). LDD was determined using Pfirrmann grading. A bespoke translational platform was designed to deliver horizontal perturbations in sagittal and frontal planes. Electromyographic activity was analysed bilaterally from 8 trunk and lower limb muscles during four established APA and CPA epochs. A Kruskal-Wallis H test with Bonferroni correction for multiple comparisons was conducted. Four groups were identified: no LDD no pain (n = 19), LDD no pain (n = 38), LDD pain (n = 35) and no LDD pain (n = 5). There were no significant differences in age or gender between groups. The most significant difference between groups was observed during forward perturbation. In the APA and CPA phases of predictable forward perturbation there were significant differences ankle strategy between groups (p = 0.007–0.008); lateral gastrocnemius and tibialis anterior activity was higher in the LDD pain than the LDD no pain group. There were no significant differences in the unpredictable condition (p>0.05). These findings were different from the remaining groups, where significant differences in hip strategy were observed during both perturbation conditions (p = 0.004–0.006). Symptomatic LDD patients exhibit different electromyographic strategies to asymptomatic LDD controls. Future LBP electromyographic research should benefit from considering assessment of both lower limbs in addition to the spine. This approach could prevent underestimation of postural control deficits and guide targeted rehabilitation.

## Introduction

Postural perturbations result from self-initiated movement or may be externally induced due to trips or slips [1], causing the centre of mass (CoM) to move close to or outside the base of support [2, 3]. This leads to system instability and subsequent loss of balance [1, 4], which may result in falls or injury if one is unable to respond effectively [3]. Translational mechanical platforms, designed to deliver external perturbations beneath the feet, are commonly used to examine stereotyped muscle responses, including anticipatory (APA) and compensatory adjustments (CPAs) [5–8]. APAs are described as the muscle activation that occur prior to a predicted perturbation event in order to minimise disequilibrium or falling [9–12], while CPAs are the muscle activations required to restore equilibrium following both predictable and unpredictable events [13, 14].

A recent systematic review suggests that when APAs and CPAs are evaluated in terms of muscle onset, people with chronic low back pain (LBP) exhibit significant muscle onset delay when compared with healthy controls in predicted and unpredicted postural perturbation conditions [15]. However, insufficient data, failure to examine both lower limb and trunk APAs and CPAs and small sample sizes, has precluded the identification of convincing differences in muscle activation or joint movement [15].

Postural perturbation strategies are usually defined in terms of muscle activation and joint movement [14]. The ‘ankle strategy’ requires the whole body to move as an inverted pendulum about the ankle joint; characterised by early activation of the dorsal ankle muscles, followed by dorsal thigh and trunk muscle activation. In contrast, the ‘hip strategy’ requires the body to move as a ‘double segment inverted with counter phase motion at the ankle and hip’ [16]; characterised by early activation of the ventral trunk and thigh muscles. In health, it has been hypothesised that the ‘ankle strategy’ and ‘hip strategy’ may be used discretely or together (‘mixed strategy’) to produce an adaptable response to postural perturbation within the sagittal plane [14]. In reality, healthy postural responses appear to represent a continuum of strategies, where strategy dominance is determined by factors including subject experience, perturbation predictability and environmental constraints [17].

Impaired postural control is often observed in those with LBP [18]. In quiet standing the ‘hip strategy’, which has been demonstrated to be more efficient in balance recovery than the ‘ankle strategy’ [19], appears restricted in people with LBP [20]. However, this increased reliance on the ‘ankle strategy’ and enhanced ankle proprioceptive acuity appears to persist during more demanding balance tasks [20–22].

Impaired LBP postural control is postulated to occur as a result of pain, fear avoidance and deficits in trunk proprioception, however, the underlying mechanisms remain unclear and need to be identified in order to direct treatment [17, 21, 23]. Although some studies have demonstrated that LBP patients exhibit different peak amplitudes and muscle activations when compared with healthy controls through the evaluation of differences in lower limb or trunk postural strategy [5, 24–26], these observations are not always consistent [22, 24, 27–30]. This reflects the heterogeneity of this patient group, for which management is challenging and remains undefined.

In advance of defining a targeted rehabilitation approach, it is important to understand the impact of LBP through the examination of postural strategies in larger, more homogeneous cohorts. Lumbar Disc Degeneration (LDD) is a prevalent condition significantly associated with LBP and changes in postural control [31–37]. However, LDD is also found in asymptomatic individuals [35, 38]. Using this cohort, the aim of this study was to investigate the differences in postural control strategy between symptomatic and asymptomatic LDD patients and healthy controls through the examination of peak amplitude and integral activity of bilateral trunk and lower limb muscles, using surface electromyography (sEMG), in response to predictable and unpredictable forward perturbations delivered by an in-plane translational platform. It was hypothesised that there would be differences between the postural control strategies adopted by symptomatic LDD patients (LDD pain) and asymptomatic controls (LDD no pain) and that these strategies would be different to those adopted by the remaining groups (no LDD pain and no LDD no pain).

## Material and methods

### Participants

A total of 97 participants completed the study (43 males and 54 females, mean age 50 years (SD 12), mean body mass index (BMI) 26 kg/m^2^ (SD 5)). Participants were recruited to this cross-sectional study through local advertising and from primary and secondary care within Imperial College NHS Trust (London, UK). Each participant provided written informed consent and was recruited in accordance with strict inclusion and exclusion criteria (Table 1) between September 2015 and January 2018 (NHS Health Research Authority, London Stanmore Research Ethics Committee, reference number: 13/LO/0793).

**Table 1:** Inclusion and exclusion criteria.

	Inclusion Criteria	Exclusion Criteria
Asymptomatic Healthy Controls *(including LDD no pain and no LDD no pain groups)*	≥ 30 years No low back pain No recurrent history of low back pain No episodes of LBP lasting greater than 3 months duration	Spinal surgery Malignancy Spondylolisthesis Peripheral neuropathy with loss of sensation Systemic or spinal infection Neurological disease or balance disorder Disorders affecting pain perception Significant cardiovascular or metabolic disease Severe musculoskeletal deformity (scoliosis, osteoporosis, Paget’s disease, fracture) Spinal surgery or major surgery within three months prior to testing MRI contraindicated Perturbation contraindicated
Symptomatic Patients *(including LDD pain and no LDD pain groups)*	≥ 30 years Evidence of LDD without neural compression on MRI Recurrent low back pain (central/ unilateral) of greater than 3 months duration MRI as part of routine NHS care	

A priori, a total sample size of 64 (16 per group) was estimated for a predefined 5% level of significance and 80% power based upon trunk peak amplitudes derived by Boudreau et al. [6] (G*Power 3.1 Statistical Power Analyses, Dusseldorf, Germany).

A 3T Verio MRI scanner (Siemens Medical Systems, Erlangen, Germany) was used to acquire supine T2 weighted sagittal lumbar spine images (L1-L5/S1) (TR = 3000 ms, TE = 92ms, 15 slices, 4mm slice width with 0.5 mm gap) from healthy controls and patients. To control for bias all Modified Pfirrmann disc grades were determined by an experienced consultant radiologist, blinded to the demographics and clinical data of participants. Participants were subsequently categorised into four groups including asymptomatic healthy controls (no LDD no pain (n = 19), LDD no pain (n = 38)) and symptomatic patients (LDD pain (n = 35) and no LDD pain (n = 5)). Participants were then identified as ‘LDD’ if they had modified Pfirrmann grade of ≥ 6 at one or more lumbar levels [39] and as ‘LBP’ if they experienced recurrent LBP for ≥ 3 months duration (pain > 0 using a numerical rating scale (NRS)) [40].

Confounders including demographics (sex, age, weight, height and BMI) and pain (NRS) were recorded for each participant during the one-off visit to the laboratory. NRS measurements were recorded before the first and following the second and third perturbation to determine changes in pain related to the trial. Additional self-reported clinical outcomes included the Hospital Anxiety and Depression Scale (HADS) [41] and Short Form 36, Version 2 (SF-36) [42] to assess anxiety and depression and quality of life, respectively.

### Experimental procedures

**Fig 1:**
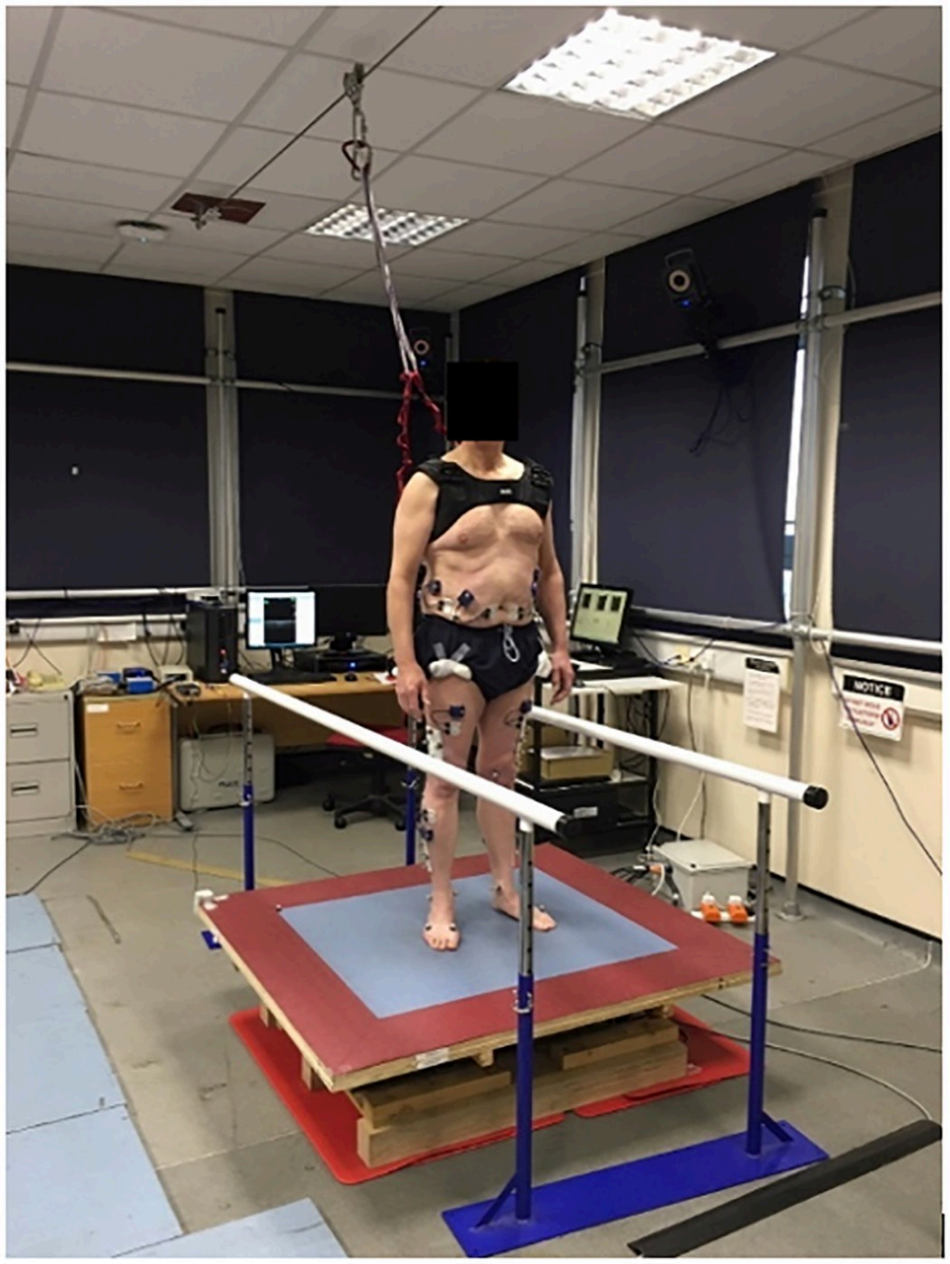
Perturbation platform and experimental set up.

A bespoke perturbation platform was designed to simulate the perturbations experienced on public transport [43]. The platform accommodated feet-in-place responses from one subject standing comfortably (Fig 1). A bespoke safety harness was worn by each participant to ensure safety and enable electrode placement without movement restriction. The ceiling was fitted with a zip wire, to which the harness was attached using a karabiner (Fig 1).

Participants were familiarised with perturbation prior to the trial by observing forward, backward, right and left perturbations of the platform itself (40 mm in 0.2 s, average acceleration 1.97 m/s^2^). A foot template and standardardised instruction encouraged the maintenance of a consistent base of support; *‘Place your feet hip width apart and maintain this position for the duration of this experiment’*. The perturbations were of sufficient magnitude to elicit compensatory feet-in-place responses without stepping. Subjects were instructed to stand barefoot in the centre of the perturbation platform with arms by the side. The safety harness was worn throughout the experiment.

In the predictable condition participants were aware of the direction and timing of each perturbation using the advanced warning provided by an audible cue five seconds in advance of the perturbation [44, 45]. In the unpredictable condition there was no auditory cues provided regarding the direction or timing of the perturbation [44, 45]. In order to limit the impact of visual cues and potential postural changes associated with visibility of perturbation graphics on the computer screen, all participants were instructed to look forward at a visual target at eye level approximately 1.5m from the platform and all perturbations were triggered with participants facing away from the computer. The conditions were presented in the same order to each subject. Subjects completed a total of three predicted and unpredicted forward, backward, left and right perturbations. Since the preliminary results indicated that the most significant difference between groups was observed during forward perturbation, this direction became the focus of our investigation.

### Electromyography

The sEMG data were acquired bilaterally from three trunk (rectus abdominis, external oblique and erector spinae) and five lower limb muscles (gluteus medius, rectus femoris, biceps femoris, tibialis anterior, lateral gastrocnemius) (Table 2) at 1000Hz using a sixteen channel system (Myon AG, Sonnenrain 75, 6103 Schwarzenburg Switzerland). The skin surface was cleaned with alcohol wipes and shaved if necessary. Disposable Ag/AgCl chloride self-adhesive sEMG electrodes (Neuroline 72000-S/25, Ambu Ltd. U.K., 2 cm inter-electrode distance) were positioned parallel to the orientation of muscle fibres in accordance with SENIAM guidelines (http://www.seniam.org) [46] and were fixed in place with surgical tape. The raw data were bandpass filtered (Butterworth filter, 10-500Hz) and rectified using MATLAB (Mathworks, Natick, MA., U.S.A.).

**Table 2:** sEMG placement.

Muscle	Abbreviation	Electrode Placement
Rectus Abdominis	RRA	Electrode positioned 3cm lateral from and inferior to the umbilicus.
	LRA	
External Abdominal Oblique	REO	Electrode positioned superior to the ASIS and lateral to the rectus abdominis, 50% on the line between the iliac crest and the ribs.
	LEO	
Erector Spinae (Longissimus)	RES	Electrode positioned two finger widths lateral of the spinous processes of L1.
	LES	
Gluteus Medius	RGMed	Electrode positioned 50% on the line between the crista iliaca and the greater Trochanter.
	LGMed	
Rectus Femoris	RRF	Electrode positioned 50% on the line between the anterior spina iliaca superior to the superior part of the patella.
	LRF	
Biceps Femoris	RBF	Electrode positioned 50% on the line between the ischial tuberosity and the lateral epicondyle of the tibia.
	LBF	
Lateral Gastrocnemius	RLGastroc	Electrode positioned 50% on the line between the head of the fibula and the heel.
	LLGastroc	
Tibialis Anterior	RTA	Electrode positioned 33% on the line between the tip of the fibula and the tip of the medial malleolus.
	LTA	

R = Right and L = left.

### Data processing

**Fig 2:**
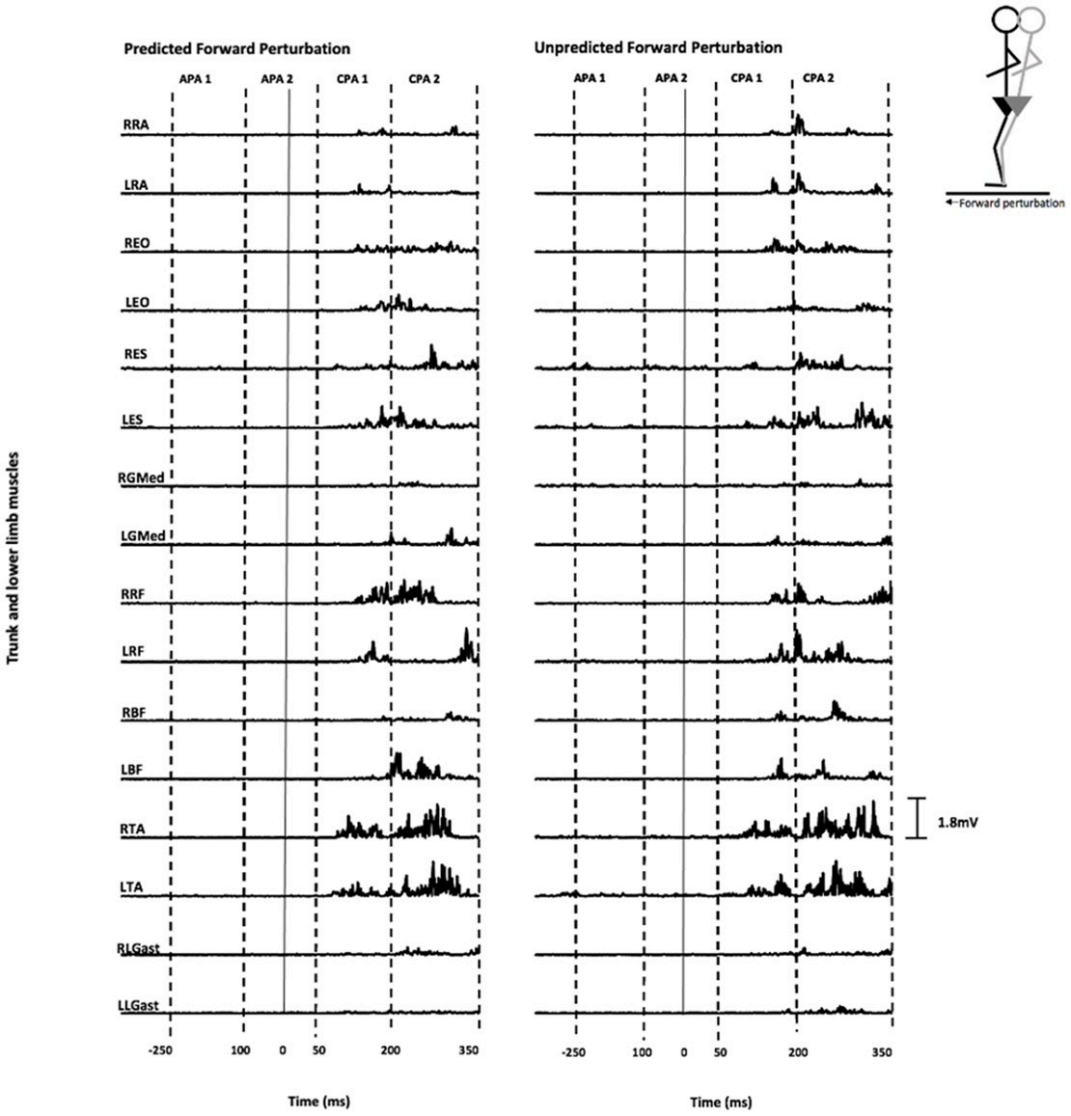
Representation of rectified sEMG trace. The original sEMG data from trunk and lower limb muscles of one healthy subject during predicted and unpredicted forward perturbation. A typical sEMG pattern averaged over three trials. 0ms represents the time of perturbation. Time scales on x axis are in ms and sEMG (mV) on the y axis. Scale (indicated bottom right) is the same for each muscle.

The sEMG signals were synchronised with the perturbation onset. The movement of the platform was assessed using an in-house constructed accelerometer (sampled at 1000Hz) attached directly to the platform. An algorithm confirmed the onset of platform perturbation using the Shewart protocol [47]. Perturbation occurred at 0ms. Differences in postural strategy were defined as a significant difference in peak sEMG amplitude following perturbation (0-350ms) or integral sEMG activation during APA and CPA time intervals defined with respect to the timing of the perturbation [1, 48]; APA1 (-250 to -100 ms), APA2 (-100 ms to +50 ms), CPA1 (+50 to 200 ms) and CPA2 (+200 to 350 ms) (Fig 2). The baseline sEMG activation was determined by calculating the mean level -500ms to -450ms prior to the perturbation (0ms) [1]. All data were corrected by subtracting the baseline activation. Three times the integral of the baseline activation was subtracted from integral sEMG activation during the APA and CPA phases since each epoch represented three times the baseline window (150ms) [1].

To enable group comparisons, the peak, integral sEMG (APA1, APA2, CPA1, CPA2) and integral sum sEMG activation (APA sum (APA1+APA2) and CPA sum (CPA1+CPA2)) were normalised to the respective maximum absolute peak amplitude, maximum integral and maximum integral sum of each muscle over 2 conditions and 3 perturbation trials for each muscle [24, 49]. Normalisation to the overall absolute maximum sEMG activity during postural perturbation tasks has been previously justified since LBP patients are often not capable or willing to generate a maximum voluntary contraction [50], as was the case in our study.

Preliminary observations concluded that peak amplitudes were consistently and significantly higher in the first perturbation trial than the second and third trials (p = 0.001–0.004). Therefore, the first of three perturbations was used for analysis.

### Statistical analysis

Statistical analysis was performed using SPSS statistical package (Version, IBM SPSS statistics, IBM Corp.). Normality was determined using QQ plots, histogram and Shapiro Wilks tests.

Values described in the results section are based upon mean ranks, since the distributions of integral sEMG activity for all postural adjustments were not the same (i.e. different shapes). This was assessed by visual inspection of a boxplot. Kruskal-Wallis H test was used to determine if there were statistically significant differences in the mean ranks of baseline sEMG activation and dependent variables (peak and integral sEMG activity (APA1, APA2, CPA1, CPA2, APA sum and CPA sum) between groups. Subsequent post-hoc analysis was performed using Dunn’s procedure [51] with a Bonferroni correction for multiple comparisons to determine group differences of the dependent variables. The effect sizes for significant comparisons of these variables were also computed ($r=\frac{z}{\sqrt{N}}$, where r = effect size, z = standard test statistic or z-score and N = total n for given comparison). The effects of confounding variables (age, BMI and sex) were examined using a Kruskal-Wallis H test and Spearman’s rho correlation coefficient (r_s_). Spearman’s rho correlations were used to explore associations between muscle activations (peak and integral EMG data) and LDD (LDD sum scores) [52], pain (NRS), anxiety and depression (HADS) and quality of life (SF-36).

Results were considered significant at P≤ 0.05. Missing data were excluded case wise from the analysis and was not replaced by imputed values. Data were also excluded in the event of protocol violation due to stepping reactions (1.85% of patient trials and 1.27% of healthy participant trials were excluded from the final analysis).

## Results

### Participants

There were no significant differences in age (p = 0.28) and sex (p = 0.58) between groups and BMI was not associated with significant findings (r_s_ = -0.20 to 0.18, p = 0.61–0.97) with one exception, right rectus abdominis in response to predicted forward perturbation (RRA APA2, r_s_ = 0.22, p = 0.04). 100% of NRS pain scores taken prior to each trial did not change following the second or third perturbation (mean scores for the LDD pain (4 (SD 3)) and no LDD pain groups (5 (SD 2)) did not change).

### Baseline activation and peak amplitude

There were no significant differences in baseline sEMG activation between groups prior to predicted and unpredicted forward perturbations in the sagittal plane (p = 0.05–0.99), apart from biceps femoris (BF) during the unpredicted perturbation (p≤0.01). This implied that in the majority of cases, any differences observed in peak amplitude and integral sEMG activity between groups were real and did not result from differences in baseline activation. There were also no significant differences in peak amplitude observed between groups following predicted (p = 0.11–0.69) or unpredicted perturbations (p = 0.25–0.93).

### Anticipatory and compensatory postural adjustments

#### Predicted forward perturbation

During predicted forward perturbation, integral sEMG activity was significantly different between groups; RLGastroc (APA2, H(3) = 10.838, p = 0.013), LTA (CPA2, H(3) = 9.643, p = 0.031), RRA (APA2, H(3) = 8.718, p = 0.033, CPA1, H(3) = 9.533, p = 0.023 and CPA sum, H(3) = 13.069, p = 0.004).

**Fig 3:**
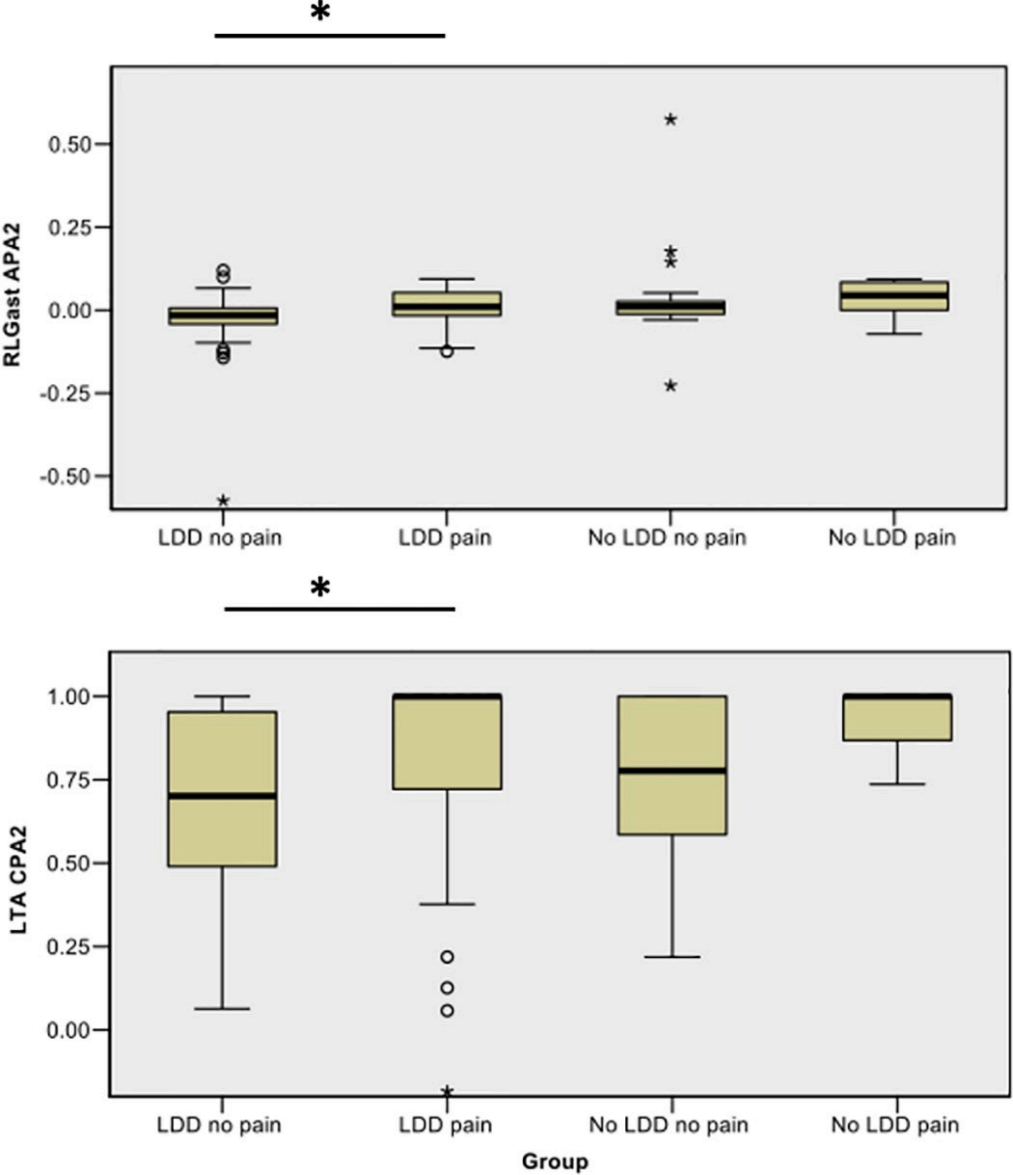
Distribution of RLGast and LTA ranks in the APA and CPA phases of predicted forward perturbation. In the APA2 and CPA2 phases of predicted forward perturbation the LDD pain group exhibited higher RLGast and LTA mean ranks than the LDD no pain group. The group names and RLGast APA2 or LTA CPA2 (integral in arbitrary units) appear on the X and Y axis respectively. The APA2 and CPA2 phases represent 150ms (-100 to 50ms (APA2) and 200 to 350ms (CPA2) post perturbation). Significance (asterisk) and individual outliers that fell 1.5 (circles) and 3 times (stars) outside of interquartile range are indicated.

Post hoc analysis revealed that during the APA phase, RLGastroc activity was higher in the LDD pain group (median rank = 55.11) than the LDD no pain group (median rank = 37.47) (RLGastroc APA2, p = 0.007, adjusted p = 0.045, medium effect size = 0.31). During the CPA phase, LTA activity was also higher in the LDD pain group (median rank = 56.70) when compared with the LDD no pain group (median rank = 39.74) (LTA CPA2, p = 0.008, adjusted p = 0.045, medium effect size = 0.31) (Fig 3).

In contrast, the remaining groups exhibited differences in trunk muscle activation. During the APA and CPA phases, RRA activity was significantly higher in the LDD no pain (median ranks APA2 = 50.46, CPA1 = 48.68 and CPA sum = 50.65) and No LDD no pain groups (median ranks APA2 = 53.63, CPA1 = 55.26 and CPA sum = 58.11) when compared to the No LDD pain group (median ranks APA2 = 11.25, CPA1 = 9.25 and CPA sum = 9.00) (adjusted p = 0.038 to 0.005, medium to large effect size = 0.42–0.69).

#### Unpredicted forward perturbation

**Fig 4:**
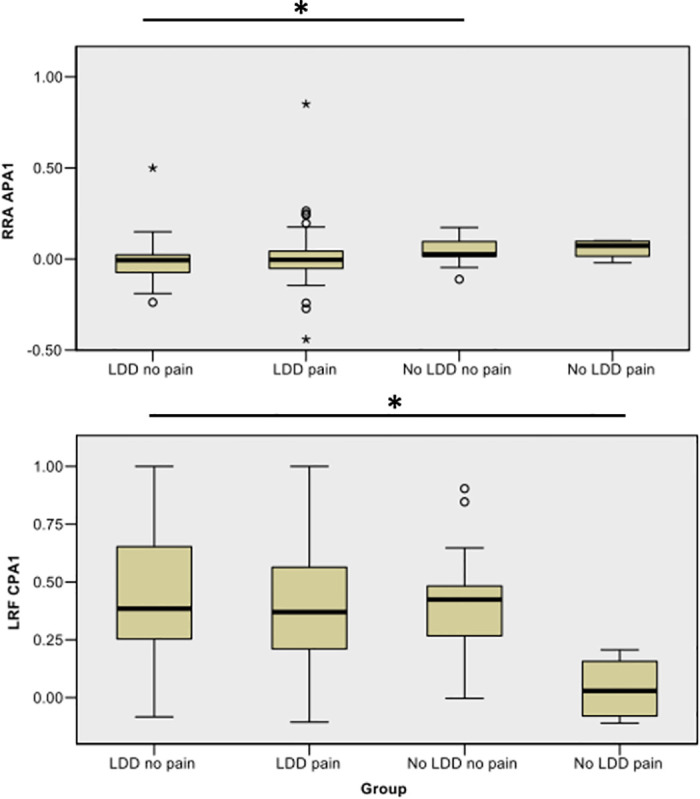
Distribution of RRA and LRF ranks in the APA and CPA phases of unpredicted forward perturbation. In the APA1 phase of unpredicted forward perturbation the LDD no pain group exhibited lower RRA mean ranks that the no LDD no pain group. In the CPA1 phase of the same condition, the LDD no pain group displayed significantly higher LRF mean ranks than no LDD pain group. The group names and RRA APA1 or LRF CPA1 (integral in arbitrary units) appear on the X and Y axis respectively. The APA1 and CPA1 phases represent 150ms (-250 to-100ms (APA1) and +50 to 200 ms (CPA1) post perturbation). Significance (asterisk) and individual outliers that fell 1.5 (circles) and 3 times (stars) outside of interquartile range are indicated.

In the unpredicted forward condition, there were no differences between the LDD pain and LDD no pain groups (p>0.05). However, integral sEMG activity was significantly different between the LDD no pain and remaining groups; LRF (CPA1, H (3) = 8.484, p = 0.037) (Fig 4) and RRA (APA1, H (3) = 9.714, p = 0.021). Post hoc analysis indicated that RRA activation was significantly lower in the LDD no pain group (median rank = 40.19) than the no LDD no pain group (median rank = 61.53) (RRA APA1, p = 0.006, adjusted p = 0.033, medium effect size = 0.37) and that LRF activity was significantly higher in the LDD no pain group (median rank = 51.46) than the no LDD pain group (median rank = 10.50) (LRF CPA1 (p = 0.004, adjusted p = 0.024, medium effect size = 0.44).

#### Associations with postural strategy

In symptomatic patients, there was no direct correlation between APAs and CPAs and self-reported pain (NRS), LDD or depression and anxiety (P>0.05). However, in symptomatic patients, anticipatory muscle activation (RLGast APA2) correlated with SF-36 items relating to pain (‘bodily pain’ r_s_ = 0.40, p = 0.02), physical functioning (‘role physical’ r_s_ = 0.45, p = 0.006, ‘vitality’ r_s_ = 0.55, p = 0.001) and mental health (‘emotional’ r_s_ = 0.49, p = 0.003, ‘mental health’ r_s_ = 0.49, p = 0.003).

## Discussion

To our knowledge, this is the first time that surface translation perturbation has been used within a larger, defined LBP cohort to determine differences in postural strategy or APAs and CPAs, as determined by muscle activation. This study provides new evidence that significant differences in postural strategy occur between LDD pain and LDD no pain groups during the APA and CPA phases of postural perturbation and that these are different to those observed in non LDD groups. This is of interest since it is known that some people with LDD experience pain (LDD pain) while others do not (LDD no pain). Therefore, understanding differences in APAs and CPAs in defined LBP cohorts could help to inform patient management.

### Peak amplitude

Preliminary observations determined that there was a significant reduction in peak sEMG amplitude in all groups over three perturbations; the highest peak amplitude was observed following the first perturbation and the lowest following the third. In the literature authors frequently describe computing the average or median response [1], but this can mask core differences. Therefore, in this study, the first of three perturbations was analysed to reflect the once off predictable and unpredictable perturbations that occur in daily life.

However, irrespective of the predictability of the perturbation, there were no significant differences in the peak sEMG amplitudes between groups. While these findings concur with a previous LBP perturbation study [53], this is in contrast with others which found decreases and increases in trunk and lower limb muscle peak activation in response to postural perturbation [24, 30, 49].

The inconsistencies presented in the literature may reflect differences in experimental protocol including normalisation procedures, baseline evaluation and the magnitude of perturbation. For example, a study which accounted for differences in baseline prior to perturbation onset and used normalisation procedures similar to that used in this current study, also found no significant differences in peak amplitudes between LBP patients and healthy controls [53]. However, in a study where significant differences in peak amplitude were found, the data did not appear to be normalised, as authors cited that LBP patients were unable to perform maximum voluntary contractions to their true maximum, which may, in part, explain this difference in results [30].

### Anticipatory and compensatory postural adjustments

Preliminary results from our previous experiments examining predicted and unpredicted right, left and backward perturbations indicated no significant difference between LDD pain and LDD no pain groups during right or leftward perturbations (p = 0.14–0.99) and where significant differences were observed, for example, during the unpredicted backward condition, the difference in muscle activation between groups, appeared to reflect a healthy, if exaggerated response, to the study protocol, which required feet-in-place responses at high acceleration [16]. Therefore, the examination of postural strategy in response to predicted and unpredicted forward perturbations became the primary focus of this study.

A recent systematic review concluded that APAs and CPAs, based upon measures of muscle onset, are delayed in response to both predicted and unpredicted perturbations in people with chronic LBP [15]. However, this review also suggested that there is no conclusive evidence that APAs and CPAs change in people with chronic LBP when measurements are based upon muscle activation [15]. This review not only highlighted a paucity of studies in this area but also a need for powered studies and comprehensive evaluation of trunk and lower limb muscle activation.

The strengths of this current study is that it is powered, larger than those described in the review [15] and it provides a comprehensive examination of both trunk and lower limb APAs and CPAs based upon muscle activation parameters. Using this approach, it has been possible to uncover significant differences in APAs and CPAs between groups. In the predicted forward perturbation condition, for example, lower limb muscle activations (RLGastroc APA2 and LTA CPA2) were higher in the LDD pain group when compared with the LDD no pain group. This response was different to asymptomatic controls (LDD no pain and No LDD no pain), who exhibited higher trunk muscle activations (RRA APA2, CPA1 and CPA sum) when compared to symptomatic LBP patients without degenerative lumbar changes (No LDD pain). In this predicted condition, the emphasis on lower limb muscle activation in the LDD pain group would appear to suggest an increased reliance on ankle APAs and CPAs in response to postural perturbation. In the absence of conclusive evidence and a paucity of studies using similar cohorts and methodologies [15], it is difficult to make direct comparison. However, in agreement with this study, people with chronic LBP have been reported to display significant differences in TA and Gastroc activations when compared to healthy controls using similarly challenging perturbations [24, 49]. This is hypothesised to be a strategic adaptation used to avoid predictably painful, multi-segmental movement [24]. In contrast, asymptomatic controls appear to rely upon increased trunk activation (RRA) when compared to symptomatic LBP patients, which reflects previous results [49].

These findings are of interest for three reasons. Firstly, in response to predicted forward perturbation, asymptomatic healthy controls (LDD no pain and No LDD no pain) appear to favour activation of the ventral trunk, which typically contributes towards the ‘hip strategy’ [16], while the LDD pain group favour lower limb activation, which may align with the hypothesis that this group is reliant upon the ‘ankle strategy’ [16]. Although further kinematic analysis is required to verify this hypothesis, these findings are of interest since the ‘trunk stiffening strategy’ [54], which is associated with LBP and fear avoidance [21, 55, 56], is reliant upon the ‘ankle strategy’ [16, 21]. Secondly, studies report that people experiencing LBP demonstrate an ankle steered proprioceptive strategy in order to retain balance [21–23], which may explain the increased TA and Gastroc activation observed in the LDD pain group. Since this ankle steered strategy increases CoM displacement following perturbation, this finding suggests that this group could be more vulnerable to instability [5, 14]. This vulnerability is further supported by evidence that symptomatic LDD patients exhibit poor postural control during static and dynamic balance tasks [36, 37]. Finally, the observation of asymmetrical significant results as well as the apparent lack of homolateral differences has been previously documented [57]. This asymmetric presentation may result from changes in axial lumbar rotation in the LDD pain group, where restricted sagittal and frontal movement, secondary to degenerative disc changes, typically necessitates increased spinal rotation [58, 59]. Although further trunk and lower limb kinematic analysis would be required to confirm this, spinal rotation in symptomatic LDD patients is likely to have consequences for the entire kinematic chain.

Overall, the predicted response appears in alignment with the evidence that people with LBP avoid pain provoking postures more than healthy controls in predicted conditions [60]. However, such results are in contrast to those observed in the unpredicted condition; there was no significant difference in muscle activation between the LDD pain and LDD no pain groups. Therefore, it could be concluded that it is the anticipation or prediction of the perturbation event (anticipatory feedforward control) that determines the ankle steered response in symptomatic LDD patients.

Asymptomatic healthy controls also demonstrated strategic differences in muscle activation between predicted and unpredicted states. Asymptomatic groups (LDD no pain and no LDD no pain) exhibited higher trunk muscle activation (RRA APA1) than symptomatic patients without degenerative changes (no LDD pain) in the predicted condition. However, this changed to a significantly higher lower limb reliance in the unpredicted scenario; the LDD no pain group demonstrated a higher lower limb activations (LRF CPA1) and lower trunk activations (RRA APA1) than the no LDD pain and no LDD no pain group respectively. Therefore, in this study, asymptomatic healthy controls demonstrate the capacity for flexible activation of proximal muscles (RRA and LRF) depending on the context of the task. It is possible that such proximal muscle activation represents a difference in ‘hip strategy’, which is typically restricted in patients with chronic LBP [17]. Since the activation patterns of asymptomatic healthy controls (proximal muscle activation) are different to symptomatic patients (distal muscle activation), it appears that the identification of phenotypes is possible in this cohort using this perturbation paradigm.

### Associations with postural strategy

Correlation analysis revealed a lack of direct association between APAs and CPAs and the degree of LDD or self-reported pain, anxiety and depression experienced by symptomatic patients. However, in the predicted condition, lower limb APAs (RLGastroc APA2) were found to be directly associated with SF-36 items relating to bodily pain, mental health and physical functioning in symptomatic patients. It is noteworthy that this is the exact muscle activation parameter (RLGastroc APA2) that was observed to be significantly different between the LDD pain and LDD no pain groups in the same condition.

The association between muscle activation and ‘bodily pain’ in symptomatic patients is important as it indicates the possibility that a NRS may not be sufficient in terms of determining the true impact of LBP. In fact, it seems that when patients are invited to report their pain experience over a longer time period (four weeks) and to reflect upon how much pain interferes with their lives that it becomes feasible to uncover the association between pain and postural strategy. It is possible that this correlation reflects a change in the ‘central set’ or the preparatory state of the CNS in response to predictable perturbations, proposed to result from an increased arousal of the nervous system due to pain or pain related fear avoidance movement in patients with active LBP [28].

### Clinical relevance

The findings of this current study suggest that differences in postural responses between LDD pain and LDD no pain groups are similar to the differences found between non-specific LBP and healthy subjects and may prove modifiable. While motor retraining has the capacity to change postural strategy [61–64], understanding specific impairments in motor control may assist the advancement of targeted treatment design and improve patient outcomes.

It has been demonstrated in this study that the response to postural perturbation depends on the predictability of the task. In the unpredictable condition, LDD pain patients moved similarly to the LDD no pain group because they could not predict the event. However, in predicted conditions LDD pain patients use maladaptive strategies, which have been found to be associated with pain and quality of life in this current study, which could increase the likelihood of falls or injury in this group by increasing susceptibility to centre of mass displacement secondary to reliance on the ankle strategy.

The observation that changes in postural strategy do not occur exclusively in the trunk and that changes may be observed in the lower limbs, suggests that a comprehensive assessment of the trunk and bilateral lower limbs is important. This approach may avoid potential underestimation of motor control impairment and treatment efficacy in future [65–67].

### Limitations

In this study a priori testing confirmed that a total sample size of 64 subjects (16 per group) would be required to obtain sufficient power. This estimate was overachieved by recruiting 97 subjects with at least 19 subjects per group (no LDD no pain = 19, LDD no pain = 38, LDD pain = 35). However, despite ongoing recruitment, it was not possible to recruit more than 5 subjects to the ‘no LDD pain’ group, indicating the rarity of symptomatic adults (>30 years) without degenerative lumbar change. Therefore, results pertaining to this group should be interpreted with caution and are not generalisable.

It is accepted that the differences in muscle activation strategy observed in this study do not reflect differences in kinematic strategy, nor do they provide information regarding co-contraction. Therefore, future analysis will be required to elucidate this. Although it could be considered a limitation that the ‘unpredictable’ condition in this study potentially underestimated differences, as participants had their eyes open throughout the trial, patient and public involvement in advance of the trial determined that patients did not feel safe and would be less likely to consent if their eyes were shut during the trial. Therefore, a suitable compromise was reached, such that participants focussed on a visual target and did not receive visual prompts or audible cues.

It is acknowledged that causation cannot be implied from this study. Therefore, it is not possible to confirm whether differences in postural control result from degenerative change, changes in reflex response modulation by higher centres, changes within the cerebral cortex (or subcortical structures) or represent difficulty in interpreting feedback. Further longitudinal research will improve understanding of these processes.

## Conclusions

LDD patients with chronic pain exhibit different postural strategies from asymptomatic LDD controls. These strategies are associated with task predictability, different to those exhibited between other groups and are not restricted to the lumbar spine. Acknowledgement of such maladaptive strategies, through the comprehensive evaluation of the spine in addition to the lower limbs, may prevent future underestimation of deficits in postural control and guide targeted rehabilitation. Further kinematic analysis will be required to understand how LBP impacts upon movement selection strategy in LDD patients.
